# Transcriptomics-driven metabolic pathway analysis reveals similar alterations in lipid metabolism in mouse MASH model and human

**DOI:** 10.1038/s43856-024-00465-3

**Published:** 2024-03-05

**Authors:** Sofia Tsouka, Pavitra Kumar, Patcharamon Seubnooch, Katrin Freiburghaus, Marie St-Pierre, Jean-François Dufour, Mojgan Masoodi

**Affiliations:** 1 Institute of Clinical Chemistry, Inselspital, Bern University Hospital, Bern, Switzerland; 2 Department for BioMedical Research, Visceral Surgery and Medicine, University of Bern, Bern, Switzerland; 3 Present Address: Centre des Maladie Digestives, Lausanne, Switzerland

**Keywords:** Non-alcoholic steatohepatitis, Metabolomics, Computational biology and bioinformatics

## Abstract

**Background:**

Metabolic dysfunction-associated steatotic liver disease (MASLD) is a prevalent chronic liver disease worldwide, and can rapidly progress to metabolic dysfunction-associated steatohepatitis (MASH). Accurate preclinical models and methodologies are needed to understand underlying metabolic mechanisms and develop treatment strategies. Through meta-analysis of currently proposed mouse models, we hypothesized that a diet- and chemical-induced MASH model closely resembles the observed lipid metabolism alterations in humans.

**Methods:**

We developed transcriptomics-driven metabolic pathway analysis (TDMPA), a method to aid in the evaluation of metabolic resemblance. TDMPA uses genome-scale metabolic models to calculate enzymatic reaction perturbations from gene expression data. We performed TDMPA to score and compare metabolic pathway alterations in MASH mouse models to human MASH signatures. We used an already-established WD+CCl4-induced MASH model and performed functional assays and lipidomics to confirm TDMPA findings.

**Results:**

Both human MASH and mouse models exhibit numerous altered metabolic pathways, including triglyceride biosynthesis, fatty acid beta-oxidation, bile acid biosynthesis, cholesterol metabolism, and oxidative phosphorylation. We confirm a significant reduction in mitochondrial functions and bioenergetics, as well as in acylcarnitines for the mouse model. We identify a wide range of lipid species within the most perturbed pathways predicted by TDMPA. Triglycerides, phospholipids, and bile acids are increased significantly in mouse MASH liver, confirming our initial observations.

**Conclusions:**

We introduce TDMPA, a methodology for evaluating metabolic pathway alterations in metabolic disorders. By comparing metabolic signatures that typify human MASH, we show a good metabolic resemblance of the WD+CCl4 mouse model. Our presented approach provides a valuable tool for defining metabolic space to aid experimental design for assessing metabolism.

## Plain language summary

Steatotic liver disease, in which fat accumulates in the liver, is one of the most prevalent liver diseases worldwide and it is important to develop relevant animal models to help us understand its mechanisms. We aimed to assess the suitability of animal models for studying steatotic liver disease in humans. We developed an approach that evaluates how genes affect the metabolism or the chemical reactions and processes that occur in the body. We used it to compare a mouse model of the disease with human observations. Our results showed that there are significant changes in fat and energy metabolism in the mouse model. These observations match with changes observed in humans, suggesting it is a good model for studying human disease. Our findings could advance our understanding of the disease as well as help define strategies for its treatment.

## Abstract (web summary)

Tsouka et al. compare metabolic signatures of metabolic dysfunction-associated steatohepatitis in a diet- and chemical-induced mouse model with human disease. By evaluating perturbations in enzymatic reactions via transcriptomics-driven metabolic pathway analysis, they observe similar alterations in lipid metabolism between the mouse model and humans.

## Introduction

Metabolic dysfunction-associated steatotic liver disease (MASLD, previously called NAFLD^1^) is the most prevalent liver disorder and affects approximately 25% of the world’s population^2–5^. MASLD is characterized by excessive accumulation of fat in the absence of a history of alcohol use or other liver diseases^6,7^. It can progress to metabolic dysfunction-associated steatohepatitis (MASH, previously called NASH^1^), which is a progressive disease histologically defined by the presence of hepatic fat (steatosis) with inflammation, and hepatocellular ballooning, and can lead to further liver injury, advanced fibrosis, cirrhosis, and hepatocellular carcinoma^8–10^. The underlying mechanisms for the development and progression of the disease are complex and multifactorial, and a ‘multiple-hit hypothesis’ has been proposed for its prediction^11^. Increased lipid synthesis and accumulation, mainly characterized by an excess of triacylglycerides (TGs), has been reported to occur from augmented de novo lipogenesis and insulin-resistant adipose tissue^12^. In turn, persistent steatosis can lead to lipotoxicity, mitochondrial dysfunction, and reactive oxygen species (ROS)-induced activation of immune cells^13,14^. Associated pro-inflammatory signaling and metabolic imbalances further aggravate the liver, leading to apoptosis, scaring, and fibrosis.

To elucidate the underlying pathophysiological mechanisms of MASLD and MASH, appropriate experimental models need to be developed. Additionally, preclinical disease models are essential for drug discovery and testing. However, due to the multifactorial nature of steatohepatitis and its heterogeneity in humans, it is challenging to establish a preclinical model that closely resembles its pathophysiology and metabolism. Several dietary (nutritional), chemical (toxin-induced), and genetic mouse models or combinations thereof have been established^15–17^. Dietary mouse models include nutrient-deficient approaches such as methionine and choline-deficient diets (MCD), and high-fat diets (HFD). HFD-induced MASLD models with the addition of fructose and/or cholesterol (also called western diet (WD)) lead to a wide spectrum of conditions that closely resemble human MASLD pathophysiology, such as insulin resistance, liver inflammation, and fibrosis^18–20^. However, HFD mouse models require a fair amount of time to develop the desired histological characteristics and often require a larger sample size to account for inter-individual response variability^21,22^. Genetically modified mouse models usually imply a loss-of-function though a gene knockout, and are often obesogenic, a known risk factor for liver disease^23^. Finally, chemically induced liver damage and fibrosis are typically used in the study of hepatic fibrosis progression and regression. However, hepatotoxins often do not reflect the usual phenotype of MASH, but rather some aspects of it, unless accompanied by a HFD^24,25^. These animal models are severe, and commonly progress to cirrhosis and hepatocellular carcinoma (HCC)^26^. Carbon tetrachloride (CCl4) has been shown to decrease induction time when combined with WD and lead to extensive liver inflammation and fibrosis^27^. The findings of this study showed that a CCl4-supplemented WD-induced MASH in only 12 weeks, and histologically matched human MASH very well. The WD provides a suitable background for inducing obesity, insulin resistance, and MASLD in mice, which are important risk factors for the development of MASH. CCl4 then acts as a “fibrosis accelerator”, allowing for much faster MASH development, while maintaining the natural course of liver metabolism.

Lipid metabolism plays a pivotal role in MASLD/MASH development and progression, thus targeting specific related enzymes/pathways provides a good strategy for drug development. Metabolomics and lipidomics approaches to investigate such mechanisms have been widely applied^12,28^. In addition, high throughput RNA sequencing has been broadly used to elucidate key mechanisms and pathways typifying MASLD and MASH progression^29–32^. A reliable method for the functional interpretation of the transcriptome is data integration in mechanistic models and subsequent simulations. Genome-scale metabolic models (GEMs) integrate all known genetic and biochemical information about an organism, effectively describing its metabolism in a network reconstruction^33^. These models can be used to perform simulations using constraint-based approaches^34^, or as a knowledge base of genes and pathways in conjunction with experimental data^35^. Over the past years, GEMs have been successfully used in MASLD research, modeling the metabolism of the human liver in health and disease^36,37^. Multiple methods to integrate transcriptomics data in GEMs have been proposed and extensively reviewed^38,39^.

Apart from the long-existing questions regarding disease development, progression, and treatment, there is an urgent need for the systematic and accurate evaluation of proposed preclinical models. In this study, we present a complete workflow to examine and compare the changes in metabolic pathways that occur across various stages of MASLD and MASH in humans, as well as in a spectrum of different mouse models. To evaluate and interpret these changes, we developed transcriptomics-driven metabolic pathway analysis (TDMPA), a method that calculates enzymatic reaction perturbations from gene expression data and uses it to score metabolic pathway alterations. To demonstrate the capabilities of TDMPA, we selected a MASH mouse model that resembles closely the human pathophysiology and used it to investigate the metabolic alterations that occur in MASH. We performed a systematic evaluation of gene expression through genome-wide RNA sequencing using liver tissues collected from mice fed a standard chow diet (controls) and the WD+CCl4 mouse model. Using the gene expression data, we scored and ranked the metabolic pathways typifying MASH, and used them as a guide to define the metabolic space related to the disease. We compared our results to human metabolic signatures and confirmed that they are in good agreement. Based on our findings, we performed further lipidomics/ metabolomics and functional analyses and confirmed enzymatic activities within the identified metabolic pathways. In the future, TDMPA can be used as a platform to evaluate the suitability of preclinical animal models and identify the most perturbed metabolic pathways, as well as to study differential gene expression on the enzymatic reaction level of the metabolic network, thus offering increased granularity and insight on the study of disease.

## Methods

### Mouse model description

Eight-week-old male C57Bl6/J mice (Charles River, Freiburg, Germany) were housed as five animals per cage under controlled temperature (22 ± 2 °C) and 12 h light-dark cycles. Mice were acclimatized to the housing facility for one week. 26 mice were randomly assigned to control (*n* = 9) or WD (*n* = 17) group and fed the respective diets for 18 weeks. The WD group was injected intraperitoneally with CCl4 (0.32 mg/kg) every week. The WD includes 42% kcal/fat, sucrose, and 1.25% cholesterol (catalog number TD.120528, Envigo Teklad). Mice’s health, body weight, and food intake were monitored weekly. Mice were weighed, anesthetized with pentobarbital (100 mg/kg, i.p.), and euthanized in the afternoon. The right lobe of the liver for each mouse was collected and stored with RNA stabilization solution (Sigma-aldrich) at −80 °C or fixed in 4% formalin. All experiments were conducted according to the regulations of the Bern Animal Welfare Committee, Canton of Bern, Switzerland (BE42/19). Histological analysis was performed by a liver-specialized pathologist to confirm induced MASH. The tissues were stained using standard H&E, oil red o, and Sirius red staining protocols. The histology images were generated using a Panoramic 250 Flash II slide scanner with a 20x objective (3DHISTECH Ltd.). Activity of ALT and AST, and total cholesterol were measured using Cobas analyzer-8000 (Roche Diagnostics GmbH, Mannheim, Germany).

### Tissue lysis and immunoblot analysis

Livers were homogenized in RIPA buffer (150 mM NaCl, 1% NP-40, 0.5% Na-deoxycholate, 0.1% SDS, and 50 mM Tris-HCl pH 7.4) containing protease and phosphatase inhibitors (Roche, Rotkreuz, Switzerland). Protein concentration was measured with the Pierce^TM^ BCA assay (Thermo Fisher Scientific, Rockford, IL, USA). Equal amounts of proteins were separated by sodium dodecyl sulfate-polyacrylamide gel electrophoresis (SDS-PAGE) and transferred to nitrocellulose membranes, blocked for 1 h with 5% nonfat milk or BSA, then incubated overnight at 4 °C with primary antibodies. After incubation with peroxidase-conjugated secondary antibody (Thermo Fisher Scientific, Rockford, IL, USA), signals were revealed with enhanced chemiluminescence (Amersham ECL Prime, GE Healthcare, Glattburg, Switzerland) and a Fusion CCD camera coupled to a computer equipped with Fusion Capt Fx Software (Vilber-Lourmat, Marne-la-Vallée, France). Signals were quantified with the Bio-1D Advanced software (Vilber-Lourmat). Uncropped and unprocessed scans of the blots are supplied as Supplementary Figs. in the Supplementary Information.

### RNA sequencing

Total RNA purification from liver tissues was performed using the RNeasy® Mini kit (Qiagen). The tissues were removed from the RNA stabilization solution, weighed, and homogenized in a buffer. The liver lysate was centrifuged, and the supernatant was collected. Ethanol was added to bind the RNA to the RNeasy membrane, the contaminants were washed away and the RNA was eluted with RNAse-free water. Transcriptomics analysis was performed using bulk RNA barcoding and sequencing (BRB-seq)^40^. The RNA was reverse transcribed with specific barcoded oligo-dT primers. The samples were pooled and the cDNA was purified using the DNA clean and concentrator kit, treated with exonuclease I, and subjected to second-strand synthesis to generate double-stranded cDNA. The full-length double-stranded cDNA was purified and eluted with water. The sequencing libraries were prepared by tagmentation of full-length double-stranded cDNA, purification with DNA clean and concentrator kit, and then elution with water. Afterward, the tagmented library was amplified, and the size of fragments 200–1000 bp was selected and sequenced using the Illumina NextSeq 500 platform.

### Lipidomics analysis

Liver tissues were homogenized in 150 mM ammonium bicarbonate by a Tissue lyser (MM300 Tissue Lyser Mixer Mill, Retsch) with a metal bead at a speed of 25 Hz for 2.5 min. The total protein content was assessed by BCA protein assay (Bio-Rad). The homogenates were extracted by methyl tert-butyl ether/ methanol (7:2) containing an internal standard (IS) mixture^41,42^. The IS mixture contained 100 pmol of DG 17:0/17:0, 1 nmol Cholesterol D7, 50 pmol of PG 17:0/17:0, 50 pmol of PA 17:0/17:0, 50 pmol of PS 17:0/17:0, 50 pmol of LPC 12:0, 50 pmol of LPS 13:0, 50 pmol of LPG 17:1, 50 pmol of LPE 17:1, 200 pmol of SM 18:1;2/12:0, 500 pmol of PC 17:0/17:0, 200 pmol of PE 17:0/17:0, 50 pmol of Cer 18:1;2/17:0, 100 pmol of TG 17:0/17:0/17:0, 200 pmol of CE 17:0. After extraction, 20 µL of the organic phase was transferred to the 96-well plate, and evaporated using a speed vacuum (SpeedVac Concentrator, Thermo Fisher Scientific). The dried samples were re-suspended in 40 µL of 7.5 mM ammonium acetate in chloroform/methanol/propanol (1:2:4, v/v/v). The lipidomics analysis was performed on Orbitrap Exploris 240 mass spectrometer (Thermo Fisher Scientific) coupled with direct infusion, a TriVersa NanoMate ion source (Advion Biosciences). The 5 µL extracted sample was directly infused into the mass spectrometer using a gas pressure of 1.25 psi and a voltage of 0.95 kV. The data was acquired in both positive and negative ionization mode in a single run. The total delivery time was set at 5 min 25 s. To avoid initial spray instability, the closure delay was set at 20 s. The polarity switched from positive to negative mode 150 s after contact closure time.

For the analysis of BAs, LPLs, and MGs, the liver homogenates were mixed with IS mixture and extracted with ice-cold methanol. The IS mixture consisted of 1 µM of CA-d5, CDCA-d4, DCA-d4, GCA-d4, GCDCA-d9, GDCA-d4, GUDCA-d4, HDCA-d5, LCA-d4, TCA-d4, TCDCA-d4, TDCA-d4, TLCA-d4, TUDCA-d4, UDCA-d4, 4 µM of LPC 17:0, LPE 17:1, and 1 µM of LPG 17:1, MG 17:0^43^. The supernatant was collected and dried under vacuum (SpeedVac Concentrator, Thermo Fisher Scientific). The dried samples were re-constituted in 40 µL of mobile phases A and B (1:1) mixture. Metabolite separation and detection were performed by ultra-high performance liquid chromatography coupled to high-resolution mass spectrometry (UHPLC-HRMS) using Vanquish UHPLC-Orbitrap Exploris 240 (Thermo Fisher Scientific). Mobile phase A was 10 mM ammonium acetate plus 0.01% acetic acid in water and mobile phase B was 10 mM ammonium acetate plus 0.01% acetic acid in methanol. The chromatographic separation was performed on an ACQUITY UPLC BEH Shield RP 18 column (Waters) with a flow rate of 0.35 mL/min. The applied ionization parameters were capillary voltage −2.5 kV, vaporization and ion transfer tube temperature set up at 300 °C, sheath gas flow rate 55 arbitrary units (AU), auxiliary gas flow rate 10 AU, and sweep gas flow rate 0 AU. The high-resolution mass spectrometry analysis was performed in negative mode with a mass range of 300-1200 m/z, running under full MS-ddMS^2^ analysis mode.

For the analysis of fatty acid oxidation and ketometabolism, the homogenates were mixed with IS and extracted with an ice-cold mixture of isopropanol: acetonitrile (1:1) and 0.1% acetic acid. The IS consisted of 1.5 µM N-acetylaspartic acid-d3, 60 µM acetoacetic acid-13C2, 5 µM decanoic acid-d19, 15 µM L-glutamic acid-d5, 2.5 µM glutaryl-L-carnitine-d3, 3 µM 3-hydroxybutyric acid-d4, 0.5 µM L-kynurenine-d4, 10 µM L-lactic acid-d3, 7.5 µM L-leucine-d3, 1 µM L-methionine-d3, 1 µM L-phenylalanine-d5, 1 µM pyroglutamic acid-d5, 0.5 µM serotonin-d4, 10 µM L-tryptophan-13C11,15N2, 20 µM threonine-13C,d2 and 8 µM L-valine-d8^44^. The supernatant was collected, evaporated under vacuum (SpeedVac Concentrator, Thermo Fisher Scientific), and re-constituted in 100 µL of mobile phase A. The analysis was performed on Vanquish UHPLC-Orbitrap Q-Exactive Plus (Thermo Fisher Scientific). The mobile system contained mobile phase A, 0.1% acetic acid in aqueous, and mobile phase B, 0.1% acetic acid in acetonitrile: isopropanol (1:1), under the flow rate of 0.35 mL/min. ACQUITY UPLC BEH C8 column (Waters) was used for the chromatographic separation. The high-resolution mass spectrometry analysis was performed in both positive and negative ionization mode, with a mass range of 85–600 *m*/*z*, running under full MS-ddMS^2^ analysis mode. The ionization parameters were as follows: capillary voltage +4.0 and −2.5 kV, heater and capillary temperature set up at 350 °C, sheath gas flow rate 45 AU, auxiliary gas flow rate 15 AU and sweep gas flow rate 1 AU.

Xcalibur software 4.4 (ThermoFisher Scientific) was used for data acquisition and spectrum preview. MS chromatograms of intact lipids were extracted by LipidXplorer software 1.2.8.1^45^. TraceFinder software 5.1 (ThermoFisher Scientific) was used for the analysis of BAs, LPLs, MGs, fatty acid oxidation, and ketometabolism.

### Respiration assay in isolated liver mitochondria

Oxygen flux was measured in liver homogenates by respirometry (Oxygraph-2k; Oroboros Instruments, Innsbruck, Austria). The 500 µg of homogenate was added to 2 mL of respiration buffer (110 mM sucrose, 60 mM K + -lactobionate, 0.5 mM EGTA, 3 mM MgCl2, 20 mM taurine, 10 mM KH2PO4, 20 mM HEPES (pH 7.1), at 37 °C). Oxidative phosphorylation was estimated with complex I (pyruvate 5 mM, malate 2 mM, glutamate 5 mM) and complex II (succinate 10 mM) substrates in the presence of ADP (2.5 mM). Leak respiration was recorded after the addition of oligomycin (2.5 µM). For maximum uncoupled respiration, the protonophore carbonyl cyanide m-chlorophenyl hydrazone (CCCP) was titrated in 0.5 µM increments until maximal stimulation of respiration. The protocol was terminated by assessing non-mitochondrial respiration with the complex I and III inhibitors, rotenone (0.5 mM), and antimycin A (2.5 mM), respectively. Finally, the activity at complex IV was recorded with the artificial substrate N,N,N’,N’-tetramethyl-p-phenylenediamine dihydrochloride (TMPD; 0.5 mM) and ascorbic acid (2 mM), and inhibited with azide (100 mM). Respiration states were corrected for non-mitochondrial respiration, and complex IV activity was corrected for azide inhibition.

### Statistical tests

For transcriptomics, RNA-Seq count data was tested for differential gene expression between control and MASH mice samples using the DESeq2 method^46^, including a Benjamini and Hochberg multiple testing correction. The adjusted *p*-value cutoff was set to 0.05 for further analysis. For lipidomics, statistical difference was assessed using the Wilcoxon two-sample test with false discovery rate (FDR) correction and considered statistically significant based on a threshold of 0.05. Missing values were imputed to 1/5 of the minimum sample value.

### Datasets used in the study

The present analysis included transcriptome data from 12 studies (six human and six mouse models). The analyzed datasets were either generated in-house (WD+CCl4 vs. CTRL) or available from literature, spanning a number of different conditions and interventions. We classified the datasets based on their respective contrast in MASLD/MASH vs. control, and MASLD/MASH stage X vs. baseline MASLD/MASH for the human cases, and in dietary, chemical, or genetic intervention vs. control for the mouse cases (Table 1). We performed analysis on a total number of 11 comparisons for human and 15 for mouse models.

**Table 1:** List of datasets used in this work.

Contrast	Cohort size	Reference	Organism
MASH F2 vs. MASLD	MASH = 153 MASLD = 53	^64^	Human
MASH F3 vs. MASLD			
MASH F3 vs. MASH F0/1			
MASH F4 vs. MASLD			
MASH F4 vs. MASH F0/1			
MASLD vs. CTRL	MASLD = 27 MASH = 25 CTRL = 39	^65^	
MASH vs. CTRL (1)			
MASLD stage 4 vs. CTRL	MASLD = 72 CTRL = 6	^66^	
MASLD stage 5 vs. CTRL			
MASLD stage 6 vs. CTRL			
MASH vs. CTRL (2)	MASH = 16 CTRL = 14	^42^	
HFD 30 vs. CTRL	HFD = 5 CTRL = 5	^65^	Mouse
MCD 8 vs. CTRL	MCD = 10 CTRL = 5		
HFD+STZ 18 vs. CTRL	HFD+STZ = 5 CTRL = 4		
WD vs. CTRL	WD = 5 CTRL = 5		
APAP vs. CTRL	APAP = 44 CTRL = 5	^66^	
CCl4 vs. CTRL (Acute)	CCl4 = 41 CTRL = 5		
CCl4 vs. CTRL (Chronic)	CCl4 = 18 CTRL = 18		
TM vs. CTRL	TM = 4 CTRL = 3		
MASH vs. CTRL (1)	MASH = 9 CTRL = 4	^68^	
MASH vs. CTRL (2)	MASH = 3 CTRL = 3	^67^	
CYP51 LKO vs. CTRL	CYP51 = 3 CTRL = 3	^69^	
GLMP KO vs. CTRL	GLMP = 4 CTRL = 4		
IKBKG LKO vs. CTRL	IKBKG = 3 CTRL = 3		
RBPJ LKO vs. CTRL	RBPJ = 3 CTRL = 4		
WD+CCl4 (MASH) vs. CTRL	MASH = 17 CTRL = 9	Present study	

Numbers after diet (HFD 30, MCD 8) correspond to number of weeks. MASLD stages 4–6 correspond to NAFLD activity score (NAS).

*MASLD* metabolic dysfunction-associated steatotic liver disease, *MASH* metabolic dysfunction-associated steatohepatitis, *CTRL* control, *SHH* sonic hedgehog, *HFD* high-fat diet, *MCD* methionine- and choline-deficient diet, *STZ* streptozocin, *WD* western diet, *APAP* acetaminophen, *CCl4* carbon tetrachloride, *TM* tunicamycin, *KO* knockout, *LKO* liver knockout, *CYP51* cytochrome P450 lanosterol 14α-demethylase, *GLMP* glycosylated lysosomal membrane protein, *IKBKG* inhibitor of kappa B kinase gamma, *RBPJ* recombination signal binding protein for immunoglobulin kappa J region.

### GEMs used in the study

In order for our results to be consistent and comparable across different organisms, we aimed to select two GEMs that are constructed on a homolog basis and are sufficiently equivalent in terms of encompassing genes, reactions, and metabolites. To this end, we selected the Human1 (version 1.12.0) and Mouse1 (version 1.3.0) metabolic models^47,48^. With the aim of making the two models equivalent, we reassigned certain reactions of the mouse model to other subsystems, namely: “MAR20010” to “Isolated”, “MAR20007” to “Purine metabolism”, “MAR20002” to “Glycosphingolipid biosynthesis-lacto and neolacto series”, and “MAR20005”,” MAR20006”, and “MAR20016” to “Transport reactions”. For additional consistency, we reassigned reaction “MAR09933” to “Acylglycerides metabolism” in both models. All of the gene identifiers provided in both GEMs and all datasets were matched using g:profiler^49^ supplemented by manual curation.

To facilitate readability, we grouped certain pathways together for presentation in the manuscript figures. These groups were defined as follows: Beta-oxidation of fatty acids: Beta-oxidation of branched-chain fatty acids (mitochondrial), Beta-oxidation of di-unsaturated fatty acids (n-6) (mitochondrial), Beta-oxidation of di-unsaturated fatty acids (n-6) (peroxisomal), Beta-oxidation of even-chain fatty acids (mitochondrial), Beta-oxidation of even-chain fatty acids (peroxisomal), Beta-oxidation of odd-chain fatty acids (mitochondrial), Beta-oxidation of odd-chain fatty acids (peroxisomal), Beta-oxidation of phytanic acid (peroxisomal), Beta-oxidation of poly-unsaturated fatty acids (mitochondrial), Beta-oxidation of unsaturated fatty acids (n-7) (mitochondrial), Beta-oxidation of unsaturated fatty acids (n-7) (peroxisomal), Beta-oxidation of unsaturated fatty acids (n-9) (mitochondrial), Beta-oxidation of unsaturated fatty acids (n-9) (peroxisomal). Carnitine shuttle: Carnitine shuttle (cytosolic), Carnitine shuttle (endoplasmic reticular), Carnitine shuttle (mitochondrial), Carnitine shuttle (peroxisomal). Cholesterol biosynthesis: Cholesterol biosynthesis 1 (Bloch pathway), Cholesterol biosynthesis 2, Cholesterol biosynthesis 3 (Kandustch-Russell pathway). Fatty acid activation: Fatty acid activation (cytosolic), Fatty acid activation (endoplasmic reticular). Fatty acid biosynthesis: Fatty acid biosynthesis, Fatty acid biosynthesis (even-chain), Fatty acid biosynthesis (odd-chain), Fatty acid biosynthesis (unsaturated). Fatty acid desaturation: Fatty acid desaturation (even-chain), Fatty acid desaturation (odd-chain). Fatty acid elongation: Fatty acid elongation (even-chain), Fatty acid elongation (odd-chain). Glycosphingolipid biosynthesis: Glycosphingolipid biosynthesis-ganglio series, Glycosphingolipid biosynthesis-globo series, Glycosphingolipid biosynthesis-lacto and neolacto series. It is important to note that TDMPA calculations and scoring were performed for each pathway separately without this grouping.

### Transcriptomics-driven metabolic pathway analysis

The first step of TDMPA is the translation of relative gene expression ratios to corresponding relative reaction flux ratios, based on the definition of gene-protein-reaction (GPR) rules, as provided in the selected GEMs. Even though absolute gene expression does not directly correlate with reaction flux for all genes, the assumption that relative changes in gene expression between two conditions correlate with changes in reaction flux profiles has been shown to perform well^50–52^. GPRs are commonly not mapped as one gene to one reaction, meaning that one gene can be potentially associated with multiple reactions and several genes can be associated with a single reaction. In the latter case, genes are mapped with “AND” and “OR” rules -or a combination thereof- to a single reaction, expressing cases of protein complexes and isoenzymes, respectively. To perform this translation we used the scheme initially introduced by Fang et al.^51^ and later also implemented in the REMI method^50^. According to this assumption, if a single gene is associated with a metabolic reaction, then the expression ratio of the gene will be assigned to the reaction ratio. If multiple genes are associated with a metabolic reaction with an “AND” rule (meaning all of them are jointly required to be expressed for the reaction to occur), then the geometric mean of the gene expression ratios is assigned as the reaction ratio. If multiple genes are associated with a metabolic reaction with an “OR” rule (meaning that the expression of any single one is sufficient for the reaction to occur), then the arithmetic mean of the gene expression ratios is assigned as the reaction ratio. If the GPR rule is a combination of the above-mentioned rules, the rule is deconstructed and the reaction ratio is calculated sequentially using the same mathematical relations. For the cases where the gene expression ratio was either missing or not significant, a value of one (or zero in the logarithmic space) was used.

After calculating the reaction ratios, we then score pathways (or subsystems as they are denoted in GEMs) based on the amount of perturbed reactions they encompass. To start with, we select a cutoff value in order to define which reactions are considered perturbed and which are not. Reactions are considered perturbed if they satisfy either ${R}_{i} > 1+{cutoff}$ or ${R}_{i} < 1/\left(1+{cutoff}\right)$, where ${R}_{i}$ is the reaction flux ratio of reaction i. In order to illustrate how this selection affects the results, we performed a sensitivity analysis on the cutoff selection (Supplementary Fig. S1— WD+CCl4 mouse model). When considering all the reaction changes larger than one percent we observed changes in almost the entire metabolic network (129 out of 141 metabolic pathways affected). The number of affected pathways dropped steeply as the cutoff was increased, namely to 96 and 57, for changes larger than 50 and 100 percent, respectively. For changes larger than 200 percent the affected pathways were only 26. Since no clear argument can be made for the choice of a larger cutoff according to our research question, we decided to keep it as low as one percent to ensure that we do not exclude any potentially valuable information.

Finally, the Pathway Reaction Score (PRS) is calculated as the percentage of perturbed reactions in the pathway, meaning the number of reactions that have an absolute computed ratio larger than the defined cutoff over the total number of reactions in the pathway. For each pathway, the PRS is calculated as:

1 $${{PRS}}_{j}=\frac{{{\#}}\, {of\; perturbed\; reactions\; in\; pathway\; j}}{{{\#}}\, {of\; total\; reactions\; in\; pathway\; j}}$$

We additionally computed *p*-values for each pathway in order to evaluate their statistical significance. We used the hypergeometric test, which is based on the hypergeometric distribution, and describes the discreet probability of k successes in m random draws without replacement, from a population of total size M that contains K objects with that attribute. In our case, and for a single pathway i, M is the total number of reactions in all pathways of the GEM, K is the number of reactions in pathway i, m is the number of reactions calculated to be perturbed based on the selected cutoff, and k is the subset of m that belongs to pathway i. The probability for the over-representation of each pathway is then calculated as:

2 $$p\left(k\right)=\frac{\left(\begin{array}{c}K\\ k\end{array}\right)\left(\begin{array}{c}M-K\\ m-k\end{array}\right)}{\left(\begin{array}{c}M\\ m\end{array}\right)}$$

where $$(\begin{array}{c}i\\ j\end{array})$$ is the binomial coefficient.

The computed *p*-values were subjected to a FDR correction, using the Benjamini and Hochberg procedure^53^.

It is usual that within one pathway some reactions are upregulated and others downregulated in disease conditions. In a number of these cases, the mean value of the pathway’s reaction fold-changes is close to zero, but the pathway is still very perturbed. Thus, it is not always straightforward to define if a pathway is up- or downregulated and to avoid the loss of information. To this end, we additionally calculated the normalized Euclidean distance for each pathway from healthy (control) conditions as a measure of pathway perturbation (Supplementary Fig. S2).

In order to evaluate the similarity between datasets, we selected Cohen’s kappa coefficient as a metric^54^. Cohen’s kappa is a statistic that is traditionally used to evaluate inter-rater reliability for categorical scales. For this, we defined three distinct categories for each reaction flux ratio as calculated by TDMPA, specifically “increased” (${R}_{i} > 1$), “decreased” (${R}_{i} < 1$), and “unchanged” (${R}_{i}=1$). Using these definitions, we constructed the confusion matrix for each metabolic pathway of the GEM, using the WD+CCl4 model as rater #1 and all the human datasets sequentially as rater #2, and then calculated Cohen’s kappa coefficient accordingly.

### Reporting summary

Further information on research design is available in the Nature Portfolio Reporting Summary linked to this article.

## Results

### Histological characterization of WD+CCl4 mouse model

**Fig. 1:**
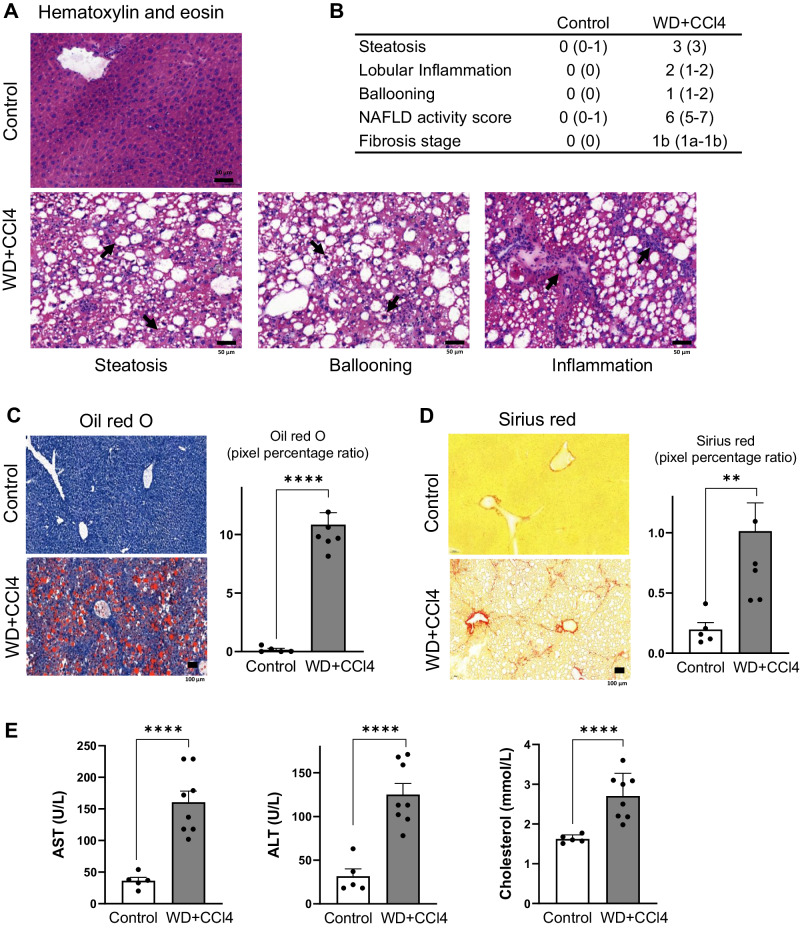
Effect of WD + CCl4 on mouse liver. **A** Microscopy of hematoxylin and eosin (H&E)-stained liver sections showing diffuse macro-vesicular steatosis, lobular inflammation, and the presence of ballooned hepatocytes in the WD+CCl4 group. **B** Histological scoring of the WD+CCl4 group livers, given as median values. Values in parentheses denote the range of values across all mice. **C** Oil Red O staining comparing neutral lipid content of control and WD+CCl4 group (unpaired *t*-test; ****p < 0.0001). **D** Microscopy of Sirius Red-stained liver sections showed increased fibrosis in WD+CCl4 group (unpaired *t*-test; **p < 0.005). **E** Liver transaminases (ALT and AST), and total cholesterol were significantly increased in WD+CCl4 group (unpaired *t*-test; ****p < 0.0001). All box plot error bars represent standard deviation. WD+CCl4: Western diet supplemented by carbon tetrachloride. (WD+CCl4 *n* = 7, control *n* = 5).

A vital first step in preclinical MASH studies is the selection of the most suitable animal model in order to address the specific research question. We decided to use a WD mouse model supplemented by CCl4 (WD+CCl4, see Methods) that accurately simulates the histological, immunological, and transcriptional characteristics of human MASH^27^. The model showed 50-fold increase in the lipid accumulation, lobular inflammation, 4-fold increase in fibrosis, and >90% micro- and macro-vesicular steatosis in the livers of MASH mice as well as the presence of ballooned hepatocytes (Fig. 1). Liver transaminases (ALT and AST), and total cholesterol were significantly elevated in the plasma of the WD+CCl4-induced MASH model compared to the control group (Fig. 1).

### Metabolic pathway alteration signatures in various stages of human MASLD/MASH progression and various liver damage mouse models

In order to assess the WD+CCl4 mouse model’s metabolic resemblance to human MASH and its suitability for metabolic intervention design, we examined the changes in metabolic pathways that occur in MASH. We investigated multiple publically available datasets covering various stages of progression of human MASLD to MASH across the full histological range from normal liver tissue to MASH with severe fibrosis (Supplementary Fig. S3). To evaluate the changes in each pathway, we used TDMPA to calculate the changes in enzymatic reactions based on differential gene expression for all datasets (see Methods). Our results suggest that different aspects of lipid metabolism are severely altered throughout the various stages of the disease. To assess the similarity of the WD+CCl4 mouse model to human MASH, we calculated Cohen’s kappa coefficient^54^ for each pathway and human dataset (see Methods). The WD+CCl4 model exhibits a good agreement with the human MASH vs. control datasets, especially with the human MASH vs. CTRL (2) dataset. This dataset corresponds to a fibrosis score of F1 (80% F1, range F0-F2), matching the fibrosis score of the mouse model. It has been proposed that Cohen’s kappa values should be interpreted as follows: values ≤ 0 as indicating no agreement, 0.01-0.20 as slight, 0.21-0.40 as fair, 0.41- 0.60 as moderate, 0.61-0.80 as substantial, and 0.81-1.00 as almost perfect agreement^55^. According to this scale, 57% of metabolic pathways between human MASH and the WD+CCl4 mouse model exhibit moderate to substantial or better agreement, including most pathways related to lipid metabolism such as acyl-CoA hydrolysis, fatty acid biosynthesis and elongation, arachidonic acid, eicosanoid, leukotriene, prostaglandin, glycerolipid, phospholipid, and sphingolipid metabolic pathways. Finally, 25% of the pathways exhibit slight agreement and 18% no agreement between the two datasets.

We additionally compared the WD+CCl4 model to other proposed mouse models spanning various intervention methods to induce liver damage (Supplementary Fig. S2). We categorized these models into three distinct classes of interventions, namely dietary, chemical, and genetic. The majority of them are in good agreement with each other and present upregulated pathways of phospholipids, nucleotides, keratan sulfate, and cholesterol esters. All models exhibit altered fatty acid oxidation, and most result in altered bile acid biosynthesis, and sphingolipid, leukotriene, and arachidonic acid metabolism. Cholesterol metabolism and biosynthesis are perturbed prevalently in the most severe dietary models. Overall, the HFD, WD, streptozotocin intoxication (STZ), and GLMP knockout mouse models exhibit the least severe metabolic changes. The model resulting in the most shifts within the metabolic network is the chronic CCl4 intoxication model, highlighting the potency of CCl4 as an acute liver damage agent.

### TDMPA shows substantial alterations in lipid metabolism in WD+CCl4 model and close resemblance to human MASH

**Fig. 2:**
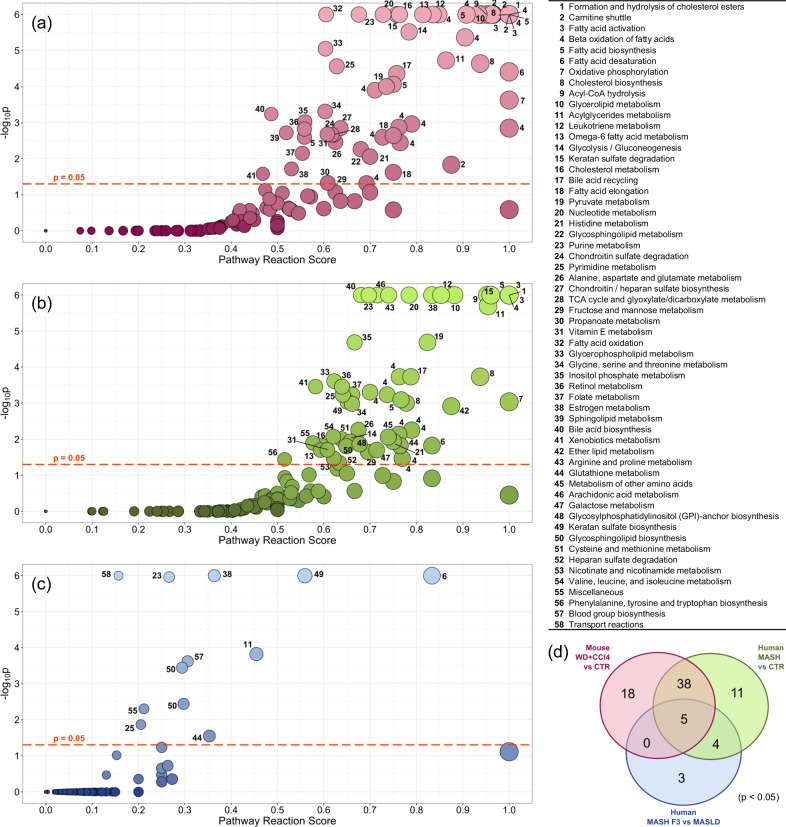
TDMPA results for the compared cases. **a** WD+CCl4 mouse model vs. Control, **b** human MASH vs. Control, **c** human MASH F3 vs. MASLD (MASH progression). **d** Corresponding Venn diagram of the statistically significant (FDR < 0.05) altered pathways across the three datasets. Number labels on the left panels (**a**, **b**, **c**) correspond to the pathways as indicated in the table on the right. WD+CCl4: Western diet supplemented by carbon tetrachloride, MASLD: Metabolic dysfunction-associated steatotic liver, MASH: Metabolic dysfunction-associated steatohepatitis.

As the next step in TDMPA, we scored metabolic pathways based on the number of altered metabolic reactions in each of them (see Methods). The results of TDMPA for the WD+CCl4 mouse model reveal multiple metabolic pathways that are significantly altered and scored highly (Fig. 2a). Most of the top-ranked pathways are related to lipid metabolism, especially fatty acids (beta-oxidation, biosynthesis, activation, desaturation, oxidation, metabolism), bile acids (biosynthesis and recycling), cholesterol (biosynthesis, metabolism, esterification), phospholipids (biosynthesis and metabolism), TGs (biosynthesis), and the carnitine shuttle, as well as oxidative phosphorylation. We performed the same analysis for human MASH F1 vs. control (corresponding to dataset MASH vs. CTRL (2) in Table 1) and MASH progression (MASH F3 vs. MASLD) datasets (Fig. 2b, c, respectively). It is clear that the mouse model matches the human MASH vs. control results very well, but is less similar to MASH progression results. This becomes even more apparent when comparing the overlap of significantly altered pathways between the three datasets (Fig. 2d). We can discern a large overlap between altered metabolic pathways in mouse and human MASH, but the resemblance of altered metabolic pathways is less in human MASH progression compared to a mouse model. Out of the identified top-scored pathways, 54% of overall changes in metabolic pathways are similar in the human MASH and WD+CCl4 mouse model. This is reduced to 11% when looking into MASLD to MASH progression. Specifically in terms of metabolic pathways, only five are commonly predicted as perturbed across all three datasets, namely fatty acid desaturation, and acylglycerides, estrogen, purine, and pyrimidine metabolism pathways. Between the mouse model and human MASH vs. control, 43 pathways are common, while 18 and 11 are only predicted in the mouse and human MASH vs. control datasets, respectively. The 43 common pathways include acyl-CoA hydrolysis, acylglycerides metabolism, bile acid biosynthesis and recycling, cholesterol biosynthesis, metabolism, and esterification, glycerolipid, glycerophospholipid, and leukotriene metabolism, glycolysis/gluconeogenesis, pyruvate metabolism, oxidative phosphorylation, estrogen metabolism, folate metabolism, purine, and pyrimidine metabolism, inositol phosphate metabolism, vitamin E metabolism, retinol metabolism, keratin sulfate degradation, the largest part of fatty acid beta-oxidation, biosynthesis, activation, and desaturation, and multiple aminoacid metabolic pathways. The mouse-only pathways include chondroitin sulfate degradation, tricarboxylic acid cycle and glyoxylate/dicarboxylate metabolism, sphingolipid metabolism, and parts of the carnitine shuttle, fatty acid biosynthesis, elongation, oxidation, and beta-oxidation. Similarly, the human MASH vs. control-only pathways include nicotinate and nicotinamide metabolism, glycosylphosphatidylinositol (GPI)-anchor biosynthesis, ether lipid metabolism, heparan sulfate degradation, and various aminoacid metabolic pathways. The MASH progression dataset results in three metabolic pathways not predicted in neither mouse nor human MASH vs. control, namely glycosphingolipid biosynthesis-lacto and neolacto series, blood group biosynthesis, and transport reactions.

**Fig. 3:**
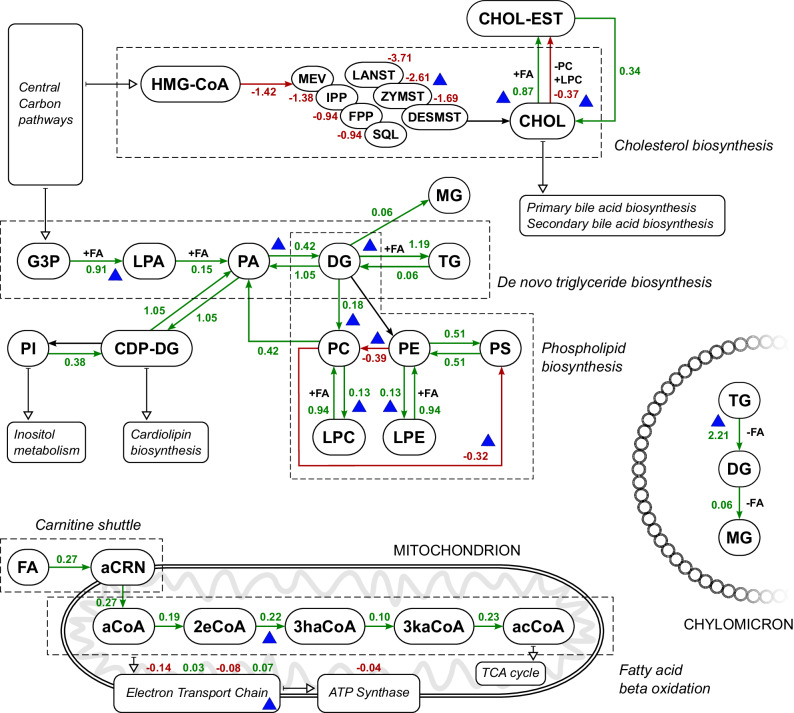
Metabolic network of the most affected pathways in mouse MASH model and corresponding reaction change ratios. Green and red colors denote a calculated increase and decrease of reaction flux, respectively. Blue triangles denote a good agreement with corresponding human data (MASH vs. CTRL (2)). Reaction change ratios are reported as log2-fold-changes. MASH Metabolic dysfunction-associated steatohepatitis, CTRL control, HMG-CoA 3-hydroxy-3-methylglutaryl coenzyme A, MEV mevalonate, IPP isopentenyl pyrophosphate, FPP farnesyl pyrophosphate, SQL squalene, LANST lanosterol, ZYMST zymosterol, DESMST desmosterol, CHOL cholesterol, CHOL-EST cholesterol ester, G3P glycerol-3-phosphate, LPA lysophosphatidic acid, PA phosphatidic acid, CDP-DG cytidine diphosphate diacylglyceride, PI phosphatidylinositol, DG diacylglyceride, TG triacylglyceride, MG monoacylglyceride, PC phosphatidylcholine, LPC lysophosphatidylcholine, PE phosphatidylethanolamine, LPE lysophosphatidylethanolamine, PS phosphatidylserine, FA fatty acid, aCRN: acylcarnitine, aCoA acyl-coenzyme A, 2eCoA 2-enoyl coenzyme A, 3haCoA 3-hydroxyacyl coenzyme A, 3kaCoA 3-ketoacyl coenzyme A, acCoA acetyl coenzyme A, TCA tricarboxylic acid, ATP: adenosine triphosphate.

Having identified the most relevant pathways, we then investigated the changes in the enzymatic reaction level (Fig. 3). Every participating reaction in de novo acylglyceride biosynthesis, especially TG biosynthesis, was upregulated. TG degradation was also increased, especially in chylomicrons. Similarly, most reactions in glycerophospholipid biosynthesis were upregulated. In addition, cholesterol biosynthesis as well as the formation and hydrolysis of cholesterol esters were severely perturbed. These results are in agreement with previous studies reporting increased de novo lipogenesis and accumulation of TGs in MASH^56^, and severely impacted cholesterol homeostasis^57,58^. Another part of the metabolic network that showed prominent changes in MASH was mitochondrial metabolism, specifically the carnitine shuttle, fatty acid beta-oxidation, and oxidative phosphorylation. We observed that the carnitine shuttle shows increased activity in MASH in both binding and transporting fatty acids to the mitochondrial matrix. Subsequently, fatty acid beta-oxidation is quite affected. Finally, oxidative phosphorylation is disrupted in both the electron transport chain and ATP synthase parts. This is in agreement with multiple reports of mitochondrial dysregulation and oxidative stress playing a substantial part in MASH^59,60^.

### Compromised mitochondrial functions and bioenergetics in MASH lead to disruption of fatty acid beta-oxidation and oxidative stress

**Fig. 4:**
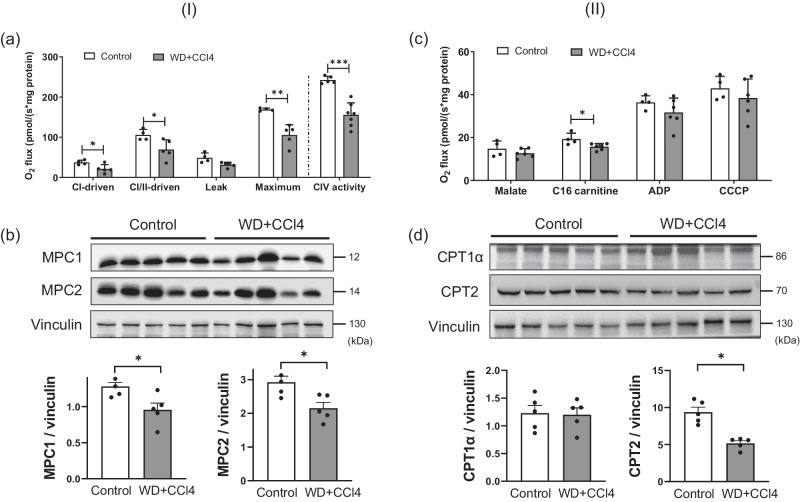
Effect of WD + CCl4 on (I) mitochondrial bioenergetics and (II) fatty acid oxidation. **a** High-resolution respirometry of oxygen consumption (O2 flux) in mitochondria in liver homogenates of control vs. WD+CCl4 group. **b** Immunoblot showing expression of mitochondrial pyruvate carrier MPC1 and MPC2 in liver homogenates. Vinculin served as the loading control. Both MPC1 and MPC2 were lower in WD+CCl4 vs. the control group. (unpaired *t*-test; *p < 0.05; **p < 0.005; ***p < 0.001). **c** fatty acid oxidation was measured by high-resolution respirometry of oxygen consumption (O2 flux) in mitochondria in liver homogenates of the control vs. WD+CCl4 group. **d** Immunoblot showing expression of carnitine palmitoyltransferase CPT1α and CPT2 in liver homogenates. Vinculin served as the loading control. CPT-2 was lower in WD+CCl4 vs. the control group. (unpaired *t*-test; *p < 0.05). All box plot error bars represent standard deviation. WD+CCl4: Western diet supplemented by carbon tetrachloride. (**a**: WD+CCl4 *n* = 7, control *n* = 5, **b**, **d**: WD+CCl4 *n* = 5, control *n* = 5, **c**: WD+CCl4 *n* = 6, control *n* = 4).

As we observed substantial alterations related to mitochondrial functions, we performed two functional assays focusing on mitochondrial bioenergetics and fatty acid oxidation in order to confirm our findings. Mitochondria isolated from the livers of MASH mice showed reduced complex I and II-driven respiration as well as maximum respiration. Complex IV was the most affected in MASH livers as shown by its independent activity measurement using the artificial substrates (TMPD+ascorbate) (Fig. 4a). Since pyruvate is the terminal glycolysis product that enters mitochondria via the mitochondrial pyruvate complex (MPC), we examined whether the expression of MPC was affected in the MASH mouse liver. In the immunoblotting assessment, both subunits of MPC were downregulated in MASH mitochondria, indicating that not only the activity of the electron transport chain (ETC) complexes was decreased significantly, but also pyruvate entry into mitochondria was lowered in MASH livers compared to control (Fig. 4b).

We additionally investigated whether fatty acid oxidation was affected in the MASH mouse livers. Respirometry provided a suitable tool to measure fatty acid oxidation since complete oxidation of a fatty acid ends in ATP generation and hence oxygen consumption via the ETC. Palmitoyl-carnitine was used as a substrate whereas malate served as a counter ion. As expected, mitochondria isolated from the liver of MASH mice had lower fatty acid oxidation compared to control mitochondria (Fig. 4c). Fatty acid uptake into the mitochondria occurs via the carnitine shuttle. Carnitine palmitoyltransferase (CPT)-1 in the outer mitochondrial membrane binds carnitine to the fatty acids to enable entry into the intermembrane space. Subsequently, CPT-2, which is located in the inner mitochondrial membrane, removes carnitine and leads the fatty acid to its oxidation in the mitochondrial matrix. Therefore, we assessed whether the expression of CPTs was affected in the MASH livers. Although the expression of CPT1α remained unchanged, CPT2 expression was reduced significantly in the MASH livers (Fig. 4d). It is interesting to note that while we confirmed a significant reduction in fatty acid beta-oxidation in the mitochondrial assay, TDMPA had predicted an increase of the pathway activity. This is due to the fact that TDMPA is a direct mapping of transcription-level changes and it does not always correspond to actual changes on the enzymatic and flux levels. However, the goal of TDMPA is to provide a first estimation of those changes and, most importantly, to direct our focus to the most affected metabolic pathways. In this respect, TDMPA correctly predicted major disruption in the mitochondrial metabolism during MASH. The occasional mismatch of the direction of change for each reaction can be attributed to cellular dynamics and molecular organization that cannot be captured solely by the transcriptome and thus needs to be accompanied by assessing enzymatic activities and functional assays.

### Metabolomics and lipidomics confirm observed metabolic alterations in MASH

**Fig. 5:**
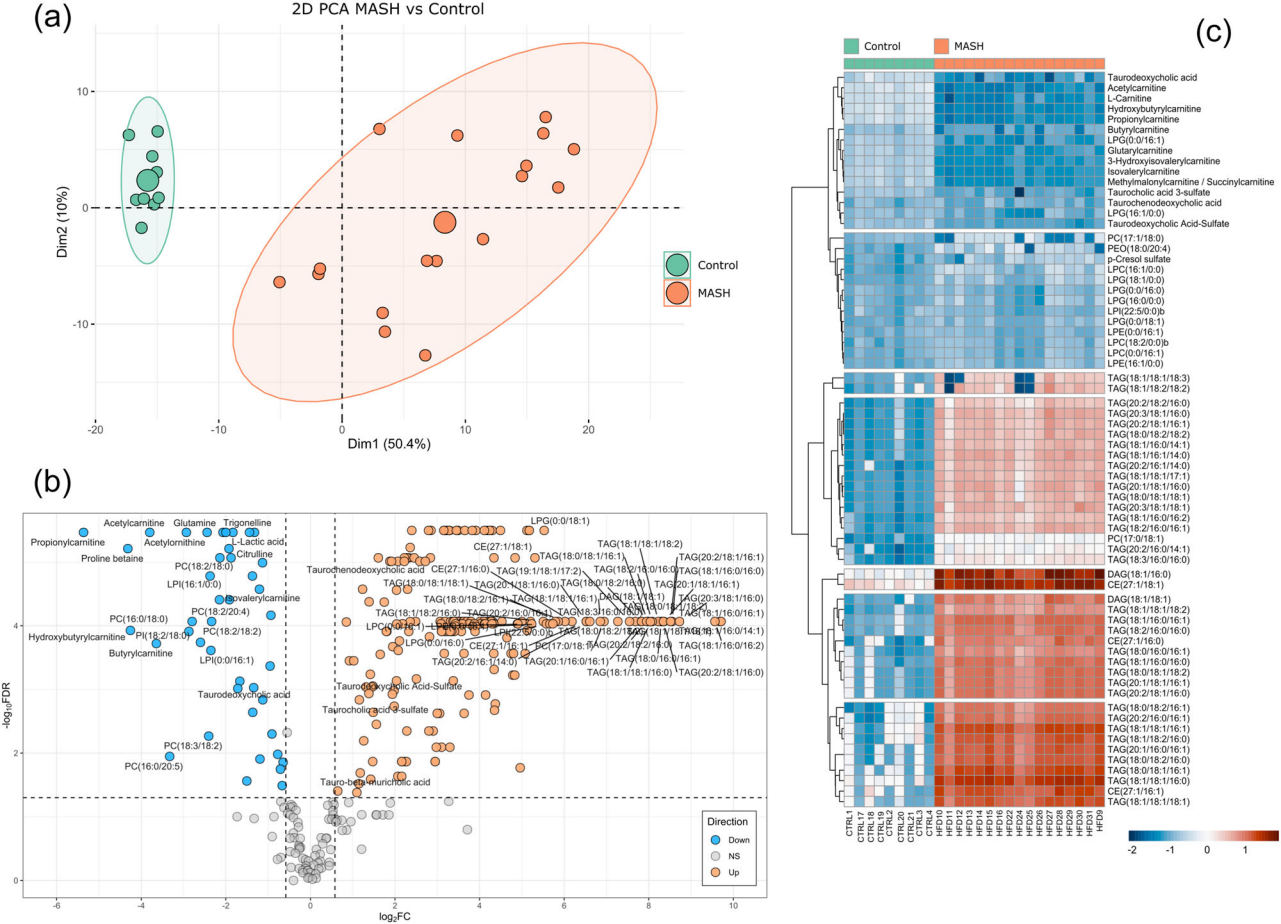
Metabolite changes in MASH vs. healthy mouse liver tissue. **a** PCA plot, **b** volcano plot, **c** heatmap of the most changed lipids. MASH metabolic dysfunction-associated steatohepatitis, PCA principal component analysis. (MASH *n* = 17, control *n* = 9).

We next evaluated the accuracy of TDMPA results to guide our investigation of the metabolic alteration occurring in MASH. We performed metabolic profiling within the targeted pathways and identified and quantified over 500 metabolites in mouse liver tissues (see Methods). In total, 252 metabolites were significantly different in the liver between control and MASH (Supplementary Data 1). Specifically, a large number of lipids were significantly different between the control and MASH groups (Fig. 5a, b). Acylcarnitines were decreased significantly in the MASH group compared to control. This is in agreement with the above-mentioned alterations in the mitochondrial beta-oxidation pathway and reduction in mitochondrial CPT2 expression. Conversely, a large number of TGs, diacylglycerides (DGs), monoacylglycerides (MGs), phosphatidylethanolamines (PEs), and cholesterol esters (CEs) were increased in MASH. Most bile acids (BAs) and lysophospholipids (LPLs) were also increased significantly in MASH. However, phosphatidylcholines (PCs) exhibited a mixed behavior. The most changes were observed in TGs, LPLs, phospholipids (PLs) (especially PEs), and acylcarnitines (Fig. 5b, c). Furthermore, we observed alterations of conjugated bile acids including taurochenodeoxycholic acid (TCDCA), taurodeoxycholic acid-sulfate (TDCA-S), taurocholic acid 3-sulfate (TCA-S), taurodeoxycholic acid (TDCA), and tauro-beta-muricholic acid (TMCA). The majority of the conjugated bile acids were significantly increased in MASH, which supports the TDMPA prediction of perturbed bile acid pathways (Fig. 2a).

**Fig. 6:**
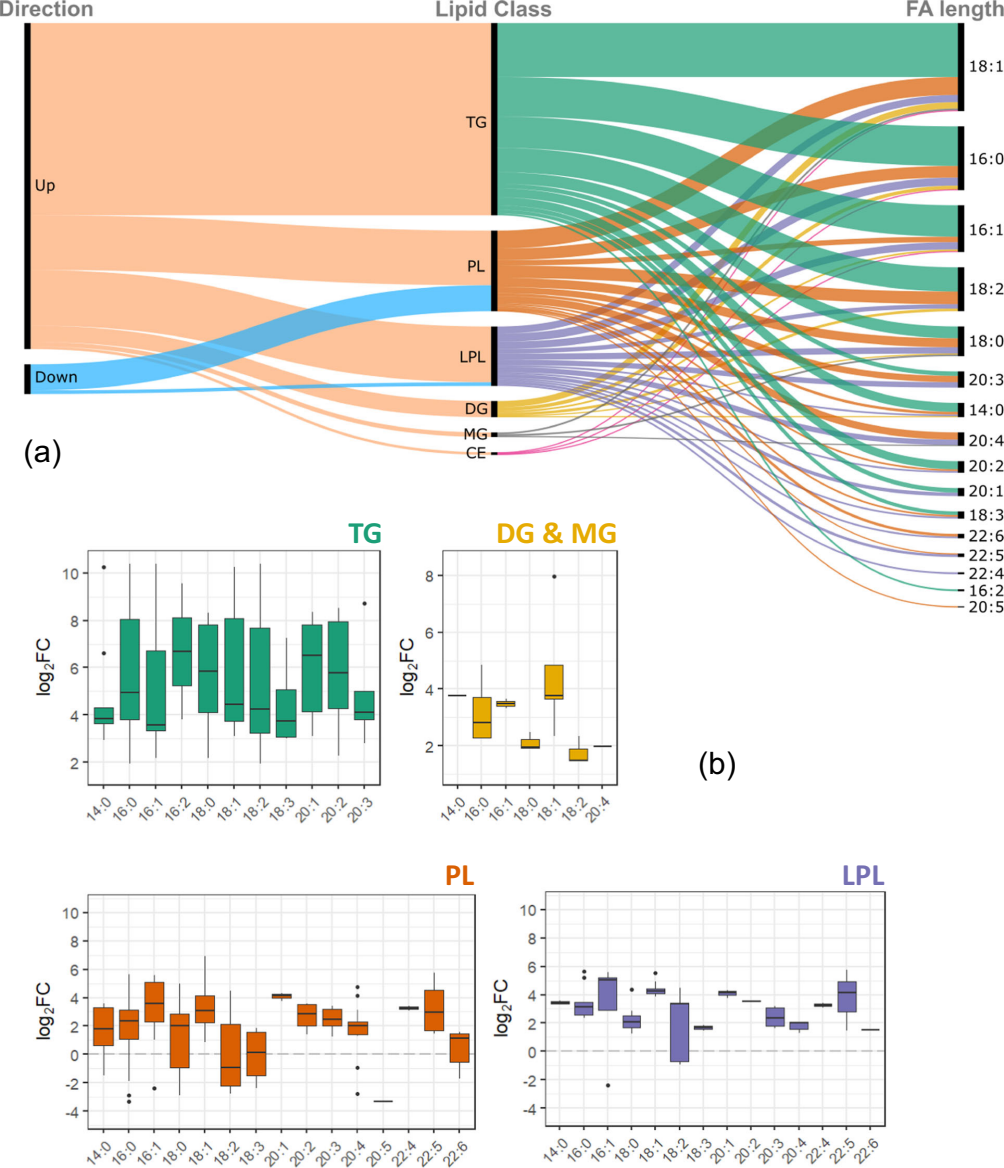
Fatty acyl chain lengths of significantly altered lipids in MASH vs. healthy mouse liver tissue. **a** Distribution per the direction of change and lipid class, **b** Box plots of log2-fold-changes per lipid class. The solid line denotes the median of the distribution and the upper and lower hinges correspond to the first and third quartiles, respectively (Tukey representation). MASH metabolic dysfunction-associated steatohepatitis, TG triacylglyceride, DG diacylglyceride, MG monoacylgyceride, PL phospholipid, LPL lysophospholipid, CE cholesterol ester. (MASH *n* = 17, control *n* = 9).

In accordance with the changes of significant lipids in MASH, we further assessed the fatty acyl (FA) chain length composition in hepatic TGs, DGs, MGs, CEs, PLs, and LPLs (Fig. 6). The majority of altered esterified FAs in MASH were saturated and monounsaturated fatty acids such as FA 18:1, FA 16:0, and FA 16:1 (Fig. 6a). Specifically, oleic acid (FA 18:1) was increased in all detected lipid species. FA 20:3, FA 20:4, FA 22:4, and FA 22:5 within PLs were also increased significantly (Fig. 6b). These fatty acids are produced via desaturation and elongation of linoleic acid. These findings support the prediction of upregulated FA desaturation and FA elongation using the TDMPA approach.

## Discussion

MASLD is one of the most prevalent liver diseases worldwide. Despite several developments in animal models, an animal model that resembles the relevant pathophysiology and metabolism needs to be established to better identify targets for treatment and drug testing. Several mouse models of MASH and their histological characterizations have been studied. However, the extent to which their metabolic alterations resemble those in humans remains poorly described. Further evaluation is needed to assess the resemblance of metabolic alterations in animal models to human conditions.

Currently, developed mouse models include dietary, chemically, and genetically induced MASLD models. HFD/WD-induced models exhibit the observed metabolic changes across the spectrum of MASLD and MASH but might require many weeks of feeding to achieve the desired histopathology^22^. Chemical intoxication leads to fibrosis much faster; however, those models typically do not result in obesity, steatosis, and insulin resistance^24,25^. Thus, these models are a good way to study physical features of liver damage and fibrosis, but potentially not very helpful to study the disease onset. A combination of WD and hepatotoxins to induce fibrosis provides a good alternative for faster disease development while preserving the phenotype^27^. We hypothesized that this approach could lead to similar metabolic changes to human MASH, thus we used the already-established mouse model to test our hypothesis. In order to evaluate the resemblance of this model to human MASH regarding metabolic alterations, we developed TDMPA using genome-scale metabolic models and transcriptomics data and further confirmed our results using functional assays, lipidomics, and metabolomics analysis. Using TDMPA, we were able to map gene expression changes to metabolic reaction rate changes, and estimate the changes across the metabolic pathways. By evaluating the highest-scoring pathways, we defined the metabolic space where most changes occur in disease development and progression, obtaining valuable insights into the metabolic fingerprint of MASH.

An essential aspect of metabolic pathway analysis is the ability to attain a certain level of granularity in the interpretation of the results, which is often lacking in gene expression analyses. We calculated the changes per metabolic reaction based on gene associations and then scored the metabolic pathways accordingly. TDMPA for the WD+CCl4-induced MASH mouse model revealed that many aspects of lipid metabolism are severely affected, which can be grouped into roughly ten pathway families for further investigation. These include bile acid biosynthesis and recycling, fatty acid beta-oxidation, biosynthesis, and metabolism, cholesterol biosynthesis, metabolism, and esterification, leukotriene and arachidonic acid metabolism, carnitine shuttle, oxidative phosphorylation, phospholipid biosynthesis, sphingolipid biosynthesis, and the metabolism of multiple amino acids. We performed the same analysis for human MASH vs. control (Fig. 2b) and MASH progression (MASH F3 vs. MASLD) datasets (Fig. 2c). We observed that the mouse model resembles the human MASH results exceptionally well when compared to control (Fig. 2a vs. b), but the resemblance was less pronounced when compared to human MASH progression results (Fig. 2a vs. c). We can discern a large overlap between the mouse and human MASH vs. control pathways, but the human MASH progression pathways are quite different from both other cases. It is very important to define well the purpose of using animal models in investigating MASH development or progression. It is worth mentioning that the focus of this study was on metabolic alterations that resemble MASH in humans.

Since we observed multiple changes in mitochondrial function, we performed two assays to evaluate the prediction. We confirmed that mitochondrial respiration and oxidation processes are disturbed in MASH mouse liver. These findings support previous hypotheses that mitochondria are heavily involved in the pathogenesis of MASLD, and could be a key player in its progression and regression^61,62^. It is important to note that TDMPA predictions in terms of the direction of changes might not always reflect actual changes in the enzymatic and flux levels, since TDMPA is a direct mapping of transcription-level changes. Nevertheless, TDMPA’s value lies in directing our focus to the most affected metabolic pathways and should be accompanied by enzymatic activity assessment and functional assays. Thus, TDMPA findings were further confirmed by lipidomics and metabolomics of the liver. More than 250 metabolites related to enzymatic reactions within the identified pathway families were significantly different in the MASH liver compared to the control. TGs were shown to be especially increased in MASH, thus further confirming the predicted increase of de novo acylglyceride biosynthesis. The increase of primary bile acids and conjugated bile acids has been reported in a previous study of MASH^63^. Further assessment of the FA chain length composition in hepatic TG, DGs, MGs, CEs, PLs, and LPLs showed an increase in saturated and monounsaturated fatty acids such as FA 18:1, FA 16:0, and FA 16:1. Specifically, oleic acid (FA 18:1) was increased in all detected lipid species.

These results taken together shed light on metabolic pathways that are commonly changed in human and selected mouse models. Our comprehensive assessment provides additional evidence that WD+CCl4-induced MASH is an appropriate mouse model for the study of human MASH pathophysiology and metabolism, confirming metabolic and functional resemblance. While TDMPA is limited by GEM annotation and exclusion of several metabolic processes such as signaling, it demonstratively offers an important level of granularity for the study of metabolic pathways in metabolic disorders and facilitates the direct mapping of changes on the gene expression level to metabolic reactions. Additionally, it is a valuable tool for defining metabolic space and better experimental design for lipidomics and metabolomics approaches in both animal models and humans. Finally, TDMPA can be used for the consistent comparison and evaluation of preclinical models, especially for targeting specific enzymes/ metabolic pathways. Accurate preclinical models and suitable methodologies to study them will enable and facilitate drug discovery and testing, identification of risk factors, development of better treatment strategies such as drug combination, and ultimately reduce the global burden of liver disease.

### Supplementary information

Supplementary information

Description of Additional Supplementary Files

Supplementary Data 1

Reporting Summary

## Data Availability

RNA-seq data files from the present study have been deposited into the Gene Expression Omnibus database (www.ncbi.nlm.nih.gov/geo/) with accession number GSE230639. Other datasets used in the study can be found in their respective original publications as listed in Table 1^42,64–69^. The list of 252 statistically significant (FDR < 0.05) lipids and metabolites can be found in Supplementary Data 1. Source data for all figures/graphs are available in Figshare (10.6084/m9.figshare.25134470.v1). All other data is available upon reasonable request from the corresponding author.

## Code availability

All in silico analyses were performed in R Statistical Software (v4.0.2; R Core Team 2020) using in-house codes. The following packages were used for visualization: “ggplot2”, “pheatmap”, “factoextra”, and “ggrepel”, and package “psych” was used to calculate Cohen’s kappa coefficient. The relevant R scripts are available upon reasonable request from the corresponding author.
