# Supplementary material for: Transcriptomics-driven metabolic pathway analysis reveals similar alterations in lipid metabolism in mouse MASH model and human
Source: Commun Med (Lond). 2024 Mar 5;4:39. doi: 10.1038/s43856-024-00465-3 (PMC10914730; doi:10.1038/s43856-024-00465-3)
Supplement: Supplementary file 1 — Supplementary information [file 43856_2024_465_MOESM1_ESM.pdf]

Supplementary Information

Transcriptomics-driven metabolic pathway analysis reveals similar alterations in lipid metabolism in mouse MASH model and human

Sofia Tsouka[1], Pavitra Kumar[2ɫ], Patcharamon Seubnooch[1ɫ], Katrin Freiburghaus[1], Marie St-Pierre[2], Jean-François Dufour[2¥], and Mojgan Masoodi[1]*

[1]Institute of Clinical Chemistry, Inselspital, Bern University Hospital, Bern, Switzerland

[2]Department for BioMedical Research, Visceral Surgery and Medicine, University of Bern, Bern, Switzerland

[¥]Current affiliation: Centre des Maladie Digestives, Lausanne, Switzerland

ɫAuthors contributed equally

* Corresponding author: Mojgan Masoodi: mojgan.masoodi@insel.ch

Supplementary description of the mouse model

The cage location and order of treatments were controlled in a palindromic sequence manner. The weight of each group was similar, to reduce the experimental error. Mice's health, body weight and food intake were monitored weekly. The different stages of the experiment (experiment implementation, and allocation period) were carried out according to the groups mentioned above. The evaluation and data analysis results were entered according to the corresponding numbers of each group. According to the allocation of different stages of the experiment, there were corresponding records on the label outside the cage. Correspondingly, the result evaluation and data analysis were analyzed according to the random cage unit. Through the experimental study, the animals were assessed by the project leader assigning scores depending on signs of immobility, loss of hunger, isolation from the rest of the group, anomalous hair, ocular opening and weight changes, according to the published guidelines[1], as follows: Weight loss: 0- Normal, 1-2- 10-20%, 3- > 20% or the animal does not drink or eat. Physical condition: 0- Normal, 1- No grooming, 2- Hair in bad condition, nasal secretions, 3- Hair in severely bad condition, anomalous stances. Behavior: 0- Normal, 1- Minor changes, weakness, 2- Abnormal: reduced mobility, inactivity, 3- Really still, comatose, vocalization, self-mutilation. If one of the single criterion reached level 3 or the animal lost more than 10% of body weight, it was euthanized by pentobarbital overdose (150mg/kg, i.p.) and exsanguination (hepatectomy). Additionally, if the sum of all the previous detailed scores was equal or higher than 4, the animal was euthanized by pentobarbital overdose (150mg/kg, i.p.) and exsanguination (hepatectomy). Finally, in the case of intermediate score (sum between 2 and 3) a subcutaneous dose of 2 mg/kg meloxicam was administered. All animals were included in the study. Body weight was used as a primary outcome measure.

For the calculation of the sample size, we used the following standard formula based on the Normal distribution[2]:

$$n = \left(Z_{a/2} + Z_{\beta}\right)^2 2 \frac{\sigma^2}{d^2} \qquad (S1)$$

, where $Z_{a/2}$ is the critical value of the Normal distribution at $a/2$, Z_{β} is the critical value of the Normal distribution at β, σ^2 is the population variance, and d is the expected difference between groups. For a confidence level of 95%, α is 0.05, and for a power of 80%, β is 0.2, resulting in critical values of 1.96 and 0.84 for $Z_{a/2}$ and Z_{β}, respectively. Thus, the factor $\left(Z_{a/2} + Z_{\beta}\right)^2$ for a confidence level of 95% and for a power of 80% equals 7.84. Based on our experience with similar animal models, the standard deviation is approximately 20%.

1. Burkholder, T., Foltz, C., Karlsson, E., Linton, C.G. & Smith, J.M. Health Evaluation of Experimental Laboratory Mice. *Curr Protoc Mouse Biol* **2**, 145-165 (2012).

2. Rosner, B. *Fundamentals of biostatistics*, (Seventh edition. Boston : Brooks/Cole, Cengage Learning, [2011] ©2011, 2011).

Supplementary figures

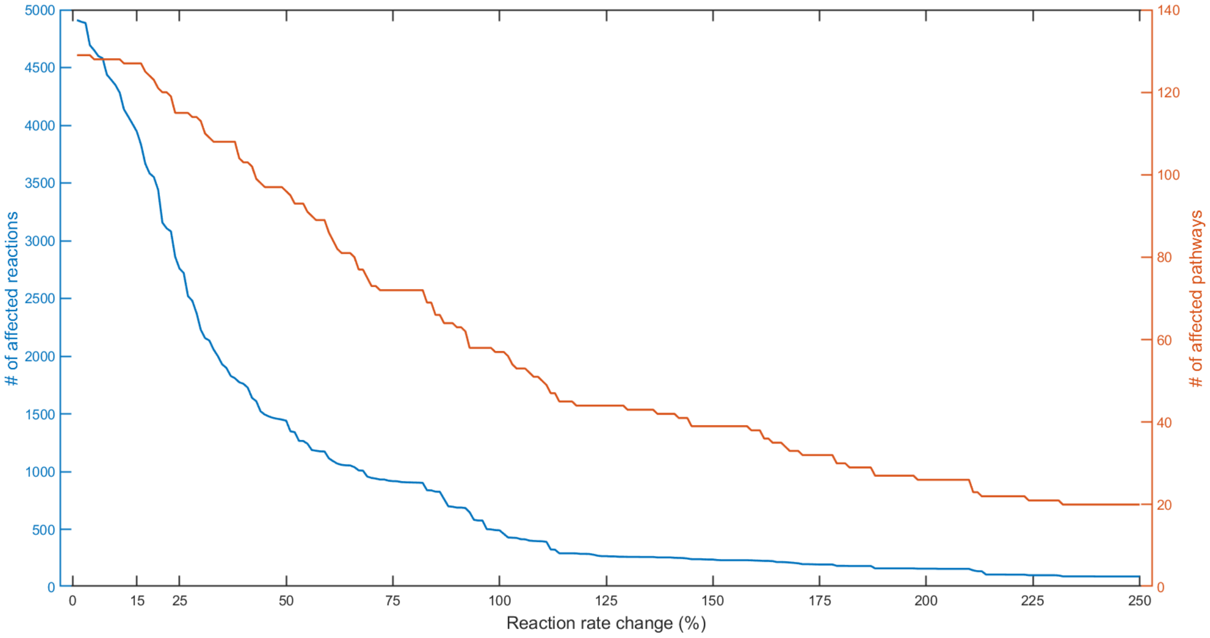

Figure S1. TDMPA sensitivity on reaction rate change cutoff.

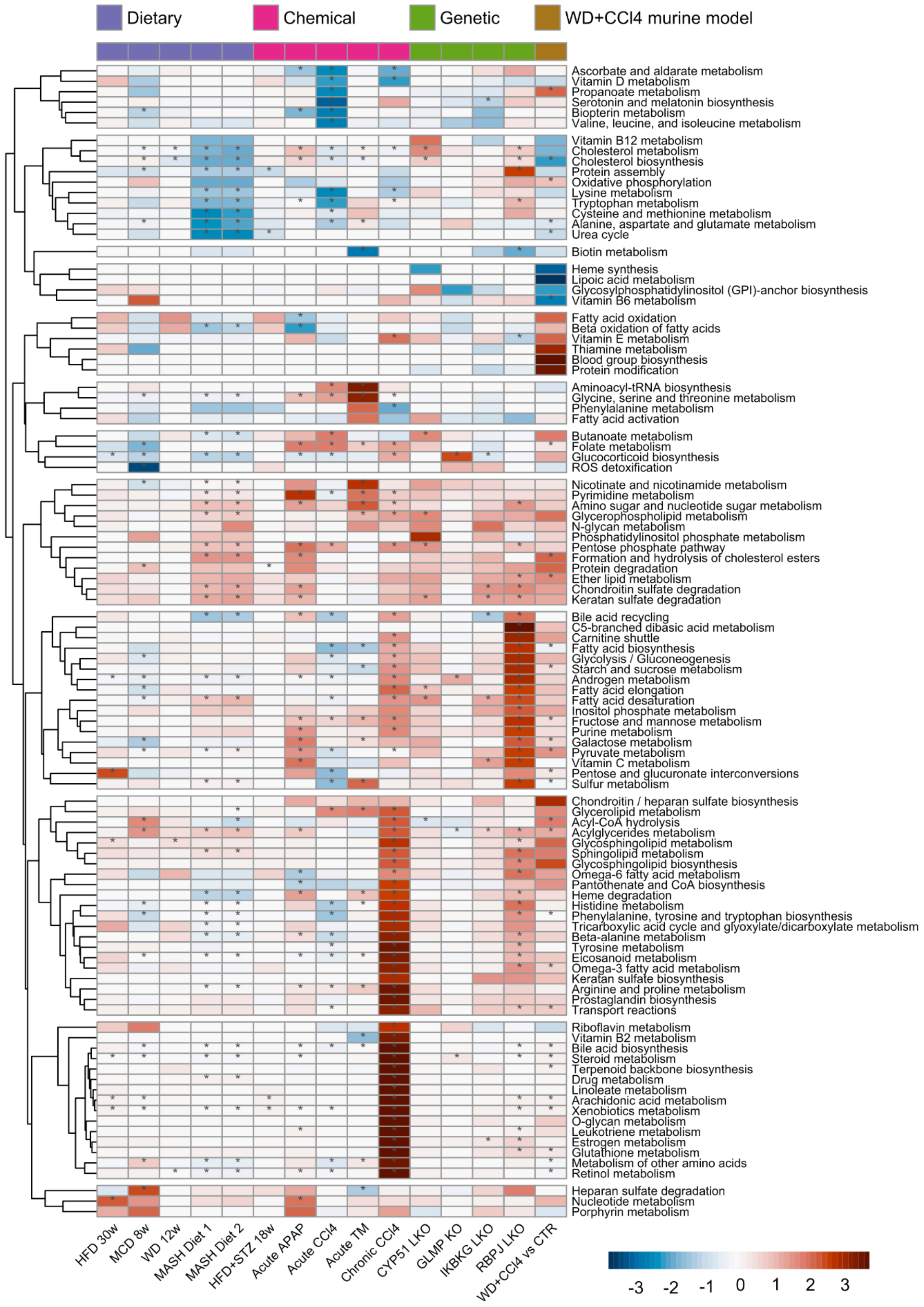

Figure S2. Comparison of pathway perturbations for murine datasets. Values are the normalized mean of the pathway's computed reaction rates. Asterisks denote the top quartile of perturbed pathways based on the normalized Euclidean distance of their reactions' rate changes from the control state.

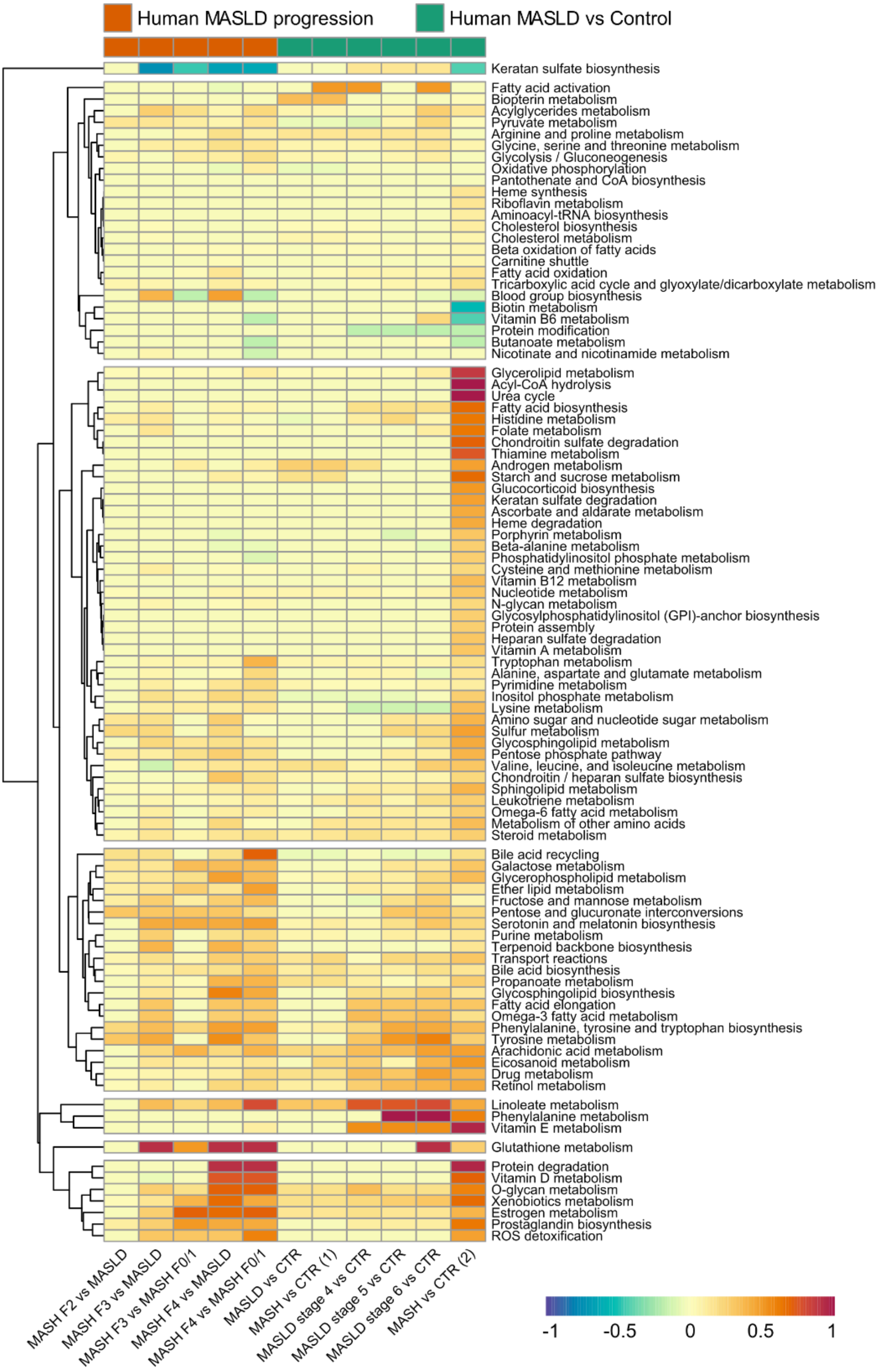

Figure S3. Evaluation of pathway perturbation similarity between human datasets and the WD+CCl4 induced MASH mouse model. Values are the Cohen's kappa coefficients calculated for each pathway.

The bands used in the figures are outlined with a dotted box.

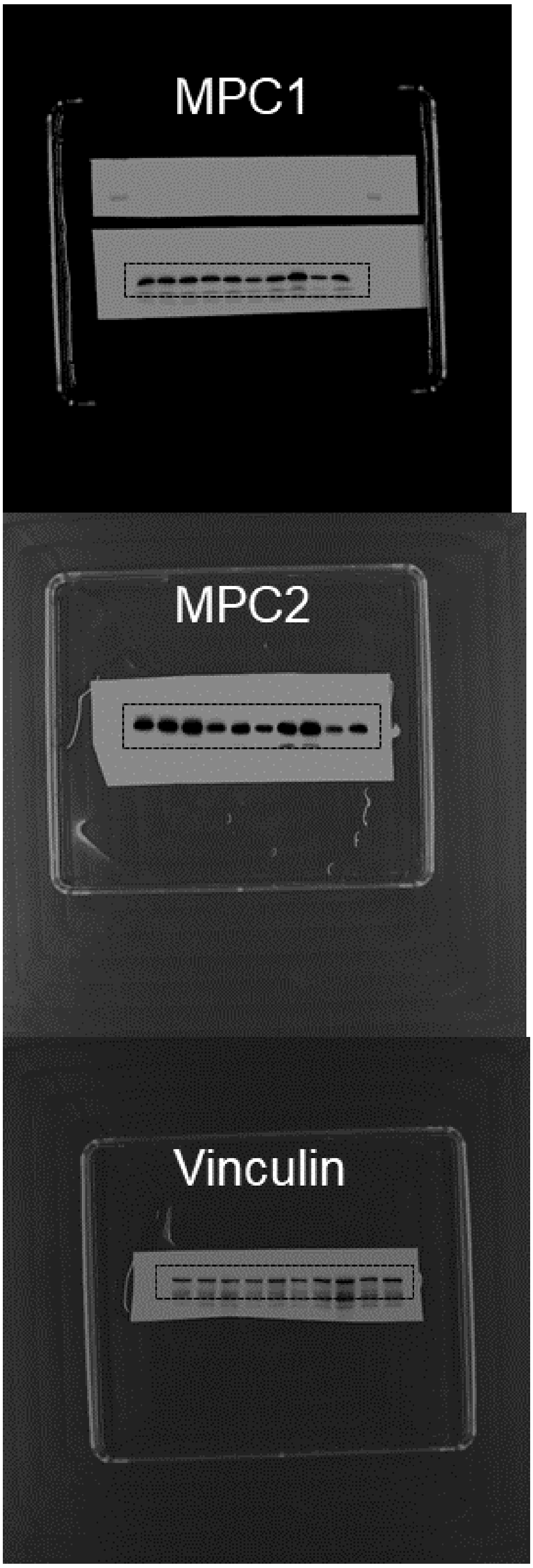

Figure S4. Uncropped blots used in Figure 4b.

The bands used in the figures are outlined with a dotted box.

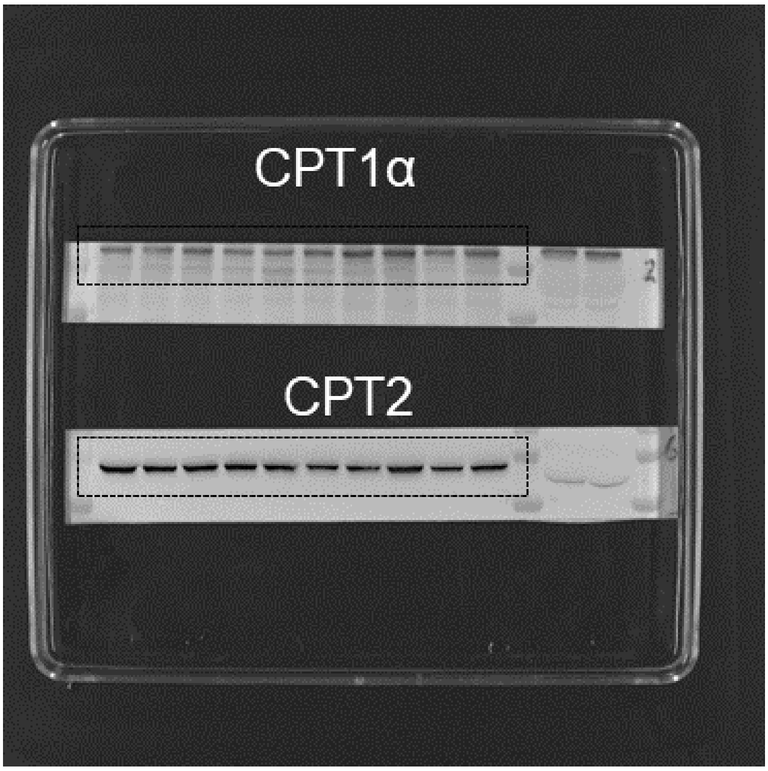

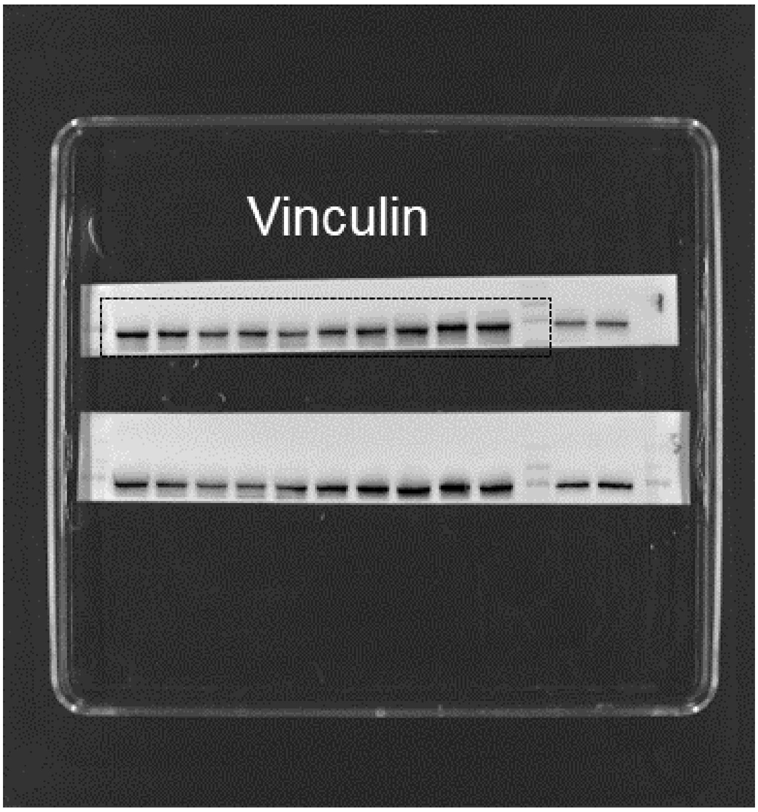

Figure S5. Uncropped blots used in Figure 4d.
