# Supplementary material for: Transcriptomics-driven metabolic pathway analysis reveals similar alterations in lipid metabolism in mouse MASH model and human
Source: Commun Med (Lond). 2024 Mar 5;4:39. doi: 10.1038/s43856-024-00465-3 (PMC10914730; doi:10.1038/s43856-024-00465-3)
Supplement: Supplementary file 2 — Description of Additional Supplementary Files [file 43856_2024_465_MOESM2_ESM.pdf]

Description of Additional Supplementary Files

File Name: Supplementary Data 1
Description: List of 252 statistically significant (FDR < 0.05) lipids and metabolites
